# Salinity Influenced Proximate, Minerals, Anti‐Nutrients and Phytochemical Composition of *Trachyandra ciliata* Kunth (Wild Cabbage): A Promising Edible Halophyte

**DOI:** 10.1002/fsn3.4755

**Published:** 2025-01-19

**Authors:** Sihle Ngxabi, Muhali Olaide Jimoh, Avela Sogoni, Charles Petrus Laubscher, Fanie Rautenbach, Learnmore Kambizi

**Affiliations:** ^1^ Department of Horticultural Sciences, Faculty of Applied Sciences Cape Peninsula University of Technology Bellville South Africa; ^2^ Department of Plant Science Olabisi Onabanjo University Ago Iwoye Nigeria

**Keywords:** Asphodelaceae, halophytes, nutraceuticals, phenolic compounds, recommended daily allowance, wild cabbage

## Abstract

Climate change, drought, and soil salinization present huge limitations to global agricultural output, which threatens food security. This necessitates the cultivation and domestication of wild edible halophytes as alternatives to mainstream food crops, especially in arid and semi‐arid regions. *Trachyandra ciliata* is one of the under‐researched and underutilized edible halophytes native to South Africa. The plant was used as a food source by Khoisan people in the past although its edibility and nutritional capacity are undocumented. Thus, the current study explored the effect of varying salinity concentrations on minerals, proximate, phytochemical, and anti‐nutrient composition of *T. ciliata* to evaluate its edibility and promote its cultivation among South African households. Plants were subjected to varying salinity treatments from 0, 50, 100,150, and 200 mM prepared by adding sodium chloride (NaCl) to the nutrient solution. Salinity significantly influenced the mineral, proximate, antinutrient, and phytochemical composition of *T. ciliata* . Control and 50 mM treatments recorded significantly higher macro and micronutrient content in the flower buds and leaves, except for heavy metals such as Zn and Cu, which increased with increasing salinity and significantly higher in the roots. Leaves under low salinity treatments recorded higher moisture and protein content, while leaves also recorded higher ash content under high salinity. On the other hand, flower buds under low salinity recorded significantly high fat and NDF composition. Phytochemicals and antinutrients increased with increasing salinity concentrations. The low antinutrient content and high nutritional, mineral and phenolic contents validate the edibility and suitability of *T. ciliata* for human consumption.

## Graphical abstract

Salinity significantly influenced the nutrients, antinutrients, and phytochemical composition of *T. ciliata* . The control and 50 mM salinity treatments significantly optimized nutrients in the flower buds and leaves. Leaves under low salinity recorded higher moisture and protein whereas high salinity optimized ash content. Flower buds under low salinity recorded significantly, high fat and NDF composition. Phytochemicals and antinutrients increased with increasing salinity. The low antinutrients and high phytonutrients in *T. ciliata* validate its edibility and suitability for human consumption.

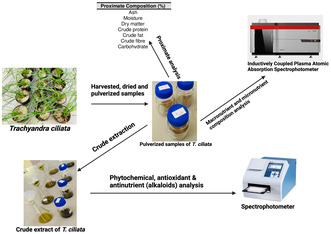

## 1 Introduction

The World Health Organization has estimated that agricultural production must rise by 50%–60% by 2050 to meet the demands of the world's constantly expanding population (Falcon, Naylor, and Shankar 2022; World Health Organization 2015). However, salinity, along with drought and other stresses, is one of the primary abiotic factors in agriculture that lead to reduced yields of edible crops worldwide (Ahanger and Agarwal 2017; Ahmad et al. 2018). Recent studies suggest that edible halophytes are excellent alternatives in some areas that are susceptible to the adverse effects of climate change due to their high tolerance to extreme temperatures, droughts, and salinity than traditional green leafy vegetable crops (Agudelo, Carvajal, and Martinez‐Ballesta 2021; Ebert 2014). Edible halophytes have developed numerous adaptation mechanisms to withstand high temperatures and salinity, and maintain nutrient and osmotic balance (Shabala 2013). In addition, developments in the food science sector and consumer preferences for varied diets point to the consumption of wild edible halophytic plants as diet complements as well as nutritious and useful foods for specific conditions. This makes commercial cultivation of these plants crucial to preventing threats from genetic erosion (Petropoulos et al. 2018).

Halophytes have also been used medicinally in various nations and are beneficial in treating and managing chronic diseases due to their high concentrations of bioactive compounds (Ngxabi et al. 2021; Petropoulos et al. 2016). Recently, these plants have been gaining momentum in Europe and Asia and are being included in restaurant menus, processed food, and beverage industries (Bvenura and Sivakumar 2017; Sogoni et al. 2021). Moreover, many of Wild edible halophytes in Asia and Europe have been utilized by rural communities as the foundation of numerous traditional cuisines and regionally significant local recipes. In contrast, in many African countries, these plants are still neglected and understudied with no scientific justification on their nutrition and edibility (Bvenura and Afolayan 2015; Petropoulos et al. 2018). Previous studies on other halophytes ( *Chenopodium quinoa* Willd, *Tetragonia decumbens* Mill, *and Chenopodium album* L.) reported that moderate salinity improved the accumulation of some essential minerals and proximate content (Rasouli et al. 2021; Sogoni et al. 2023; Sogoni et al. 2021). Sogoni et al. (2021) further reported that high salinity levels led increased phytochemical contents of *Tetragonia decumbens*. However, most of these wild edible halophytes are underutilized and understudied especially in many African countries although they have potential as alternatives to mainstream vegetable crops (Bvenura and Afolayan 2015; Ogundola, Bvenura, and Afolayan 2018).

*Trachyandra ciliata* (wild cabbage) is one of the under‐researched and underutilized halophytes native to the Western Cape coastal dunes, South Africa. It is classified under Asphodelaceae (Aloe family), which is well known worldwide for its importance in the pharmaceutical industry (Manning and Goldblatt 2007). Interestingly, this plant is reported to have been used as a food source by the Khoisan people who resided in the coastal areas of the Western Cape before colonization and massive industrialization of the province. The flower buds (which resemble the edible shoots of *Asparagus officinalis* ) were steamed and cooked as vegetable or added into stews and salads (De Vynck, Van Wyk, and Cowling 2016). However, its edibility, nutritional capacity, and pharmacological properties are undocumented and yet to be explored. A previous study by Ngxabi (2020) on *T. ciliata* reported that moderate salinity promoted growth, while recent studies on a closely related species ( *T. divaricata* ) reported the presence of essential minerals above the Recommended Daily Allowance (RDA) and high phenolic compounds (Bulawa et al. 2022; Tshayingwe et al. 2023). It is therefore imperative to study the nutritional value, phytochemical, and anti‐nutrient composition of *T. ciliata* to investigate its edibility to promote its consumption and commercialization. Findings from this study are anticipated to serve as points of reference for commercial growers, households, public, scholars, and aspiring researchers whose interests are on the potential of easily accessible underutilized vegetable crops and medicinal plants.

## 2 Materials and Methods

This research was carried out at the Cape Peninsula University of Technology's Bellville campus in Cape Town, South Africa, at coordinates 33.55048.800 S and 18.38032.700 E. Using environmental control, the study's experimental greenhouse temperatures were maintained between 21°C and 26°C during the day and between 12°C and 18°C at night, while average relative humidity was maintained at 60%.

### 2.1 Experimental Design

#### 2.1.1 Plant Material

The Cape Peninsula University of Technology nursery, Bellville campus provided the *Trachyandra ciliata* plant material. Due to the presence of rhizomes, the plant material was propagated through division technique (Bulawa et al. 2022). Once established about 150 plants were transferred in 12.5 cm pots that contained a mixture of peat, perlite and vermiculite at 1:1:1 ratio. To guarantee maximum genetic homology, cuttings were taken from the same mother stock population. The plantlets were split into five treatments, each consisting of thirty duplicates, and arranged in blocks.

#### 2.1.2 Nutrient Solutions

Nutrifeed fertilizer supplied by STARKE AYRES Pty. Ltd. Hartebeesfontein Farm, Bredell Rd., Kaalfontein, Kempton Park, Gauteng, South Africa, 1619 has been certified as containing all the essential nutrients necessary for healthy and vigorous plant growth and is now widely used to make aqueous solutions that promote maximum production of plants. The specifications are as follows: 75 mg/kg S, 240 mg/kg B, 240 mg/kg Ca, 10 mg/kg Mo, 22 mg/kg Mg, 240 mg/kg Mn, 65 g/kg N, 27 g/kg P, 130 g/kg K, 70 mg/kg Ca, 20 mg/kg Cu, 1500 mg/kg Fe, and 240 mg/kg Zn. In this experiment, fertilizer group 1 Reg No: K2025 (Act 36/1947) was used to supply to all the blocks to deliver the same amount of nutrients to the nutrient solution. For the first 4 weeks, the plants were irrigated solely with this solution before salinity was gradually increased to minimize transplant shock.

#### 2.1.3 Salinity Treatments

Different salt concentrations were established by adding sodium chloride (NaCl) to the nutritional solutions. This experiment examined four salt concentrations: 50, 100, 150, and 200 mM of NaCl. The control group (0 mM) received only nutrient solution irrigation. NaCl was gradually added to other treatments after 4 weeks of the plants being irrigated with Nutrifeed solution. Plants received 300 mL of the nutrient solution, either with or without NaCl, at equal intervals every 3 days. Drain water from each pot was gathered, and the electrical conductivity was measured to ascertain that the right salinity levels were maintained. Using a calibrated hand‐held digital pH meter (EurotechTM pH2 pen), the solution's pH was kept at 6.0. The nutrition solution's pH was raised with potassium hydroxide and lowered with phosphoric acid. A calibrated hand‐held digital electrical conductivity (EC) meter (Hanna InstrumentsTM HI 98312) was used to measure the EC in the nutrient solution every day. The plants were harvested after 15 weeks of salinity treatment to conduct the analyses.

### 2.2 Mineral Analysis of Leaves, Roots, and Flower Buds

With an Inductively Coupled Plasma‐Optical Emission Spectrometer (Varian Vista‐MPX, Victoria 3170, Australia), the mineral content of leaves, roots, and flower buds harvested from each replicate treatment was examined (Bulawa et al. 2022; Tshayingwe et al. 2023). The analyses were conducted at the KwaZulu Natal Department of Agriculture and Rural Development's mineral analysis laboratory.

### 2.3 Proximate Analysis of Leaves, Roots, and Flower Buds

#### 2.3.1 Moisture Content

To ascertain the moisture content of the studied plant samples, a method outlined by Jimoh, Afolayan, and Lewu (2020) was slightly altered. After being dried in an oven at 105°C for an hour, empty ceramic vessels were weighed and labeled W1, then allowed to cool. After being weighed out, 1 g samples of *Trachyandra ciliata* (W2) were placed in a container and dried at 105°C in an oven until a consistent weight was achieved. After being chilled in a desiccator, the container together with the plant material were weighed again (W3). The % moisture content was determined using the below formula:

$$\%\text{moisture content}=\frac{W2-W3}{W2-W1}\times 100$$

#### 2.3.2 Crude Fiber Content

The pulverized and air‐dried sample (1 g) was weighed in an empty crucible (W1). At room temperature, 100 mL of neutral detergent solution and 0.5 g of Na_2_SO_3_ was added to the crucible, followed by a few drops of octanol. Following 60 min of boiling, the mixture was filtered, and the residue was washed two times in cold acetone and hot water. The residue was then oven‐dried for 8 h at 105°C before being cooled in a desiccator and weighed (W2). The following formula was applied to calculate the Neutral Detergent Fiber (NDF) content as reported by Tshayingwe et al. (2023).

$$\%\text{Neutral Detergent Fibre}\;\left(\mathrm{NDF}\right)=\frac{\left(W1+W2\right)-W1}{\text{Weight of the sample}}\times 100$$

#### 2.3.3 Crude Fat Content

The composition of fat content of the leave, roots, and flower buds of Wild cabbage was estimated following a laboratory method as described by Cebani et al. (2024). An orbital shaker was used to agitate one gram of the ground plant with 100 mL of diethyl ether for 24 h. Following extraction, the mixture was strained, and the resulting filtrate was then poured into a freshly weighed beaker. After homogenizing the ether extract with 100 mL diethyl ether and shaking it for a further 24 h on an orbital shaker, the filtrate was collected in a beaker labeled W1. Prior to being reweighed in the beaker (W2), the ether filtrate was oven‐dried at 55°C after being dried in a steam bath to concentrate. The following equation was used to calculate the crude fat of the extracts:

$$\%\mathrm{fat}\;\text{content}=\frac{W2-W1}{\text{original weight of the pulverised sample}}\times 100$$

#### 2.3.4 Crude Ash

A procedure outlined by Tshayingwe et al. (2023) was used to assess the percentage ash content of the examined *Trachyandra ciliata* leaves, roots, and flower buds. After being labeled with sample codes using a heat‐resistant marker, porcelain crucibles were oven‐dried for 1 h at 105°C. Once the crucibles were cooled in a desiccator, they were weighed (W1). Subsequently, 1 g of plant powder was weighed and added to ceramic crucibles that had been previously pre‐weighed (W2). In order to completely ash the samples, the crucibles and their contents were heated in a muffle furnace to 250°C for an hour followed by heating to 550°C for 5 h. The samples were weighed following their cooling in a desiccator (W3).

$$\%\mathrm{ash}\;\text{content}=\frac{W2-W3}{W2-W1}\times 100$$

#### 2.3.5 Crude Protein

Using a digestion tablet as a catalyst, 2 g of each pulverized sample was boiled in 20 mL of concentrated H_2_SO_4_ in a Kjeldahl flask to produce a clear liquid, which was used to quantify crude protein. After being filtered and diluted in 250 mL, the digested extracts were then distilled. In a 500 mL round‐bottomed flask, an aliquot containing 50 mL of 45% NaOH was further distilled, and 150 mL of the distillate was put into a flask containing 100 mL of 0.1 M HCl. After that, methyl orange was used to titrate this against 2.0 mol/L NaOH. A yellow color shift signaled the titration's endpoint, and the percentage of nitrogen content was assessed using the following equation.

$$\text{Crude protein}=\frac{\left[\left(\text{mLstd acid}\times N\;\text{of acid}\right)-\left(\mathrm{mL}\;\text{bank}\times N\;\text{of base}\right)\right]-\left(\text{mLstd base}\times N\;\text{of base}\right)\times 1.4007}{\text{original weight of the pulverised sample}}\times 100$$

Where *N* = normality and the percentage of crude protein were obtained by multiplying the nitrogen value by a constant factor of 6.25 (Mndi et al. 2023).

### 2.4 Antioxidant and Phytochemical Essays

Phytochemical content and antioxidant capacity of metabolites in the plant extract were assayed for total flavonols, flavonoids, total polyphenols, ferric reducing antioxidant power (FRAP), ABTS, and DPPH.

#### 2.4.1 Formulation of Crude Extracts

The crude extracts were formulated by applying a protocol as outlined by Faber et al. (2020) The finely crushed leaves, roots, and flower buds harvested at week 15 were stirred in ethyl alcohol (EtOH) and centrifuged for 5 min at 4000 rpm to obtain crude extracts. A Buchner funnel with an electric vacuum pump attached to it held Whatman No. 1 filter paper, through which the supernatant was filtered. Unmacerated tissue and other debris were removed using this procedure. Phytochemical and antioxidant assays were conducted using the crude extracts that were obtained from all of the treated plant samples.

#### 2.4.2 Total Polyphenol Assay

The Folin–Ciocalteu technique was used to analyze the extracts' total polyphenol content, as detailed by Sogoni et al. (2021). About 125 μL of Folin–Ciocalteu reagent (Merck, Johannesburg, South Africa) was diluted ten times with distilled water and combined with about 25 μL each of the crude extract. Subsequently, a 96‐well microplate containing the extract was filled with a 7.5% sodium carbonate (Sigma, Alberton, South Africa) solution. The absorbance of the plate was measured at 765 nm using a Multiskan spectrum plate reader (Thermo Electron Corporation, Waltham, MA, USA) after it was incubated for 2 h at room temperature. The results were represented as milligrams gallic acid equals per g dry weight (mg GAE/g DW), and the standard curve was created using 0, 20, 50, 100, 250, and 500 mg/L gallic acid (Sigma, South Africa) in 10% EtOH.

#### 2.4.3 Determination of Flavonol Content

The method to quantify flavonol composition was derived from a study by Faber et al. (2020). Quercetin 0, 5, 10, 20, 40, and 80 mg/L in 95% ethanol (Sigma‐Aldrich, Johannesburg, South Africa) was used as a baseline to calculate the flavonol concentration of the extracts. Quercetin equivalent milligrams per gram of dry weight, or mg QE/g DW, was used to express the results.

#### 2.4.4 Estimation of Flavonoid Content

By following the aluminum chloride spectrophotometric assay with slight modification as reported by Jimoh et al. (2023), the total flavonoid in the crude extracts and standard was determined. The assay measures the yellow‐orange coloring of the flavonoid‐AlCl_3_ complex, which results from the interaction of flavonoids with AlCl_3_. In 1 mg mL^−1^ of methanol, a stock of the plant extracts and standard (quercetin) was made. Quercetin extract solutions (0.5 mL) and graded concentrations (0.2–1.0 mg mL^−1^) have been prepared and then put into different test tubes. The test tubes were then filled with 2 mL of distilled water and 0.15 mL of 5% NaNO_2_. After completely vertexing the mixture, it was let to stand at room temperature for 6 min. After that, 0.15 mL of 10% AlCl_3_ and 1 mL of 1 M NaOH were added to the mixture and allowed to sit for 5 min. The solution was diluted with distilled water to a volume of 5 mL, and it was then incubated for 30 min at 40°C in a water bath. After incubation, the solution was transferred into a glass cuvette, and the spectrophotometer was used to detect absorbance at 430 nm. The standard calibration curve was used to quantify the total flavonoid content of the crude extract as mg g^−1^ of quercetin equivalent (QE g^−1^).

#### 2.4.5 Determination of Ferric Reducing Antioxidant Power (FRAP)

A 30 mL volume of acetate buffer (0.3 M, pH 3.6) (Merck, South Africa) was combined with 3 mL of 2,4,6‐tripyridyl‐s triazine (10 mM in 0.1 M hydrochloric acid) (Sigma, South Africa), 3 mL of iron (III) chloride hexahydrate (FeCl_3_·6H_2_O) (Sigma, South Africa), and 6 mL of distilled water to create the FRAP reagent. The mixture was then incubated for 30 min at 37°C. Subsequently, 300 μL of the FRAP reagent and 10 μL of the crude sample extract were combined in a 96‐well plate. After that, the absorbance was determined using a Multiskan spectrum plate reader at 593 nm. To determine the FRAP sample values, L‐ascorbic acid (Sigma‐Aldrich, South Africa) was utilized as a standard, with a concentration curve ranging from 0 to 100 M. The results were expressed as μM ascorbic acid equivalents (AAE) per g dry weight (μM AAE/g DW) (Ngxabi et al. 2021).

#### 2.4.6 Determination of ABTS Antioxidant Capacity

Using a technique outlined by Jimoh et al. (2023) with slight adjustments, the ABTS antioxidant capacity was measured. A 140 mM of potassium peroxide (K_2_S_2_O_8_) (Merck, South Africa) and 7 mM of ABTS were prepared as stock solutions. Following that, 5 mL of ABTS solution was mixed with 88 μL of K_2_S_2_O_8_to create the experiment's solution. These two solutions were combined, and they were allowed to react at room temperature for 24 h in the dark. The standard utilized in the experiment was Trolox (6‐Hydrox‐2,5,7,8‐ tetramethylchroman‐2‐20 carboxylic acid), which was present in quantities ranging from 0 to 500 μM. The absorbance was measured at 734 nm at 25°C in a microplate reader after crude sample extracts (25 μL) were allowed to react with 300 μL ABTS in the dark at room temperature for 5 min. Trolox equivalent per gram of dry weight (μM TE/g DW) was used to express the results.

#### 2.4.7 Antioxidant Capacity of DPPH Free Radicals

The DPPH free radical antioxidant capacity was measured by employing a protocol as outlined by (Unuofin, Otunola, and Afolayan 2017). The 0.135 mM DPPH solution, which was made in a dark bottle, produced the DPPH radical. A reaction was conducted using approximately 300 μL of DPPH solution, 25 μL of crude extract, and graded concentrations (0 and 500 μM) of Trolox standard (6‐Hydrox‐2,5,7,8‐tetramethylchroman‐2‐ 20 carboxylic acid) solution. After 30 min of incubation, the integrates' absorbance at 517 nM was measured. The results were presented as μM/Trolox equivalent per g dry weight (μM TE/g DW).

### 2.5 Determination of Anti‐Nutrients

#### 2.5.1 Quantification of Alkaloids

The total alkaloid composition of leaves, roots, and flower buds was quantified using the Bromo cresol green method based on an atropine standard as described by Faber et al. (2020). In order to extract the plant material, 100 mg was placed in 10 mL of methanol inside a 15 mL lab‐grade plastic tube. The tubes were then centrifuged for 2 min at 4000 rpm. A 1 mL of methanol extract, 5 mL of buffered solution (pH 4.7), 12 mL of bromocresol green, and 12 mL of chloroform were added to a 50 mL lab‐grade plastic tube. When the chloroform separates from the buffering solution and Bromo cresol green, alkaloids are present in the extract if a yellow color is recognized. After that, the yellow chloroform liquid would be put in a multi‐scan spectrum scanner by pipetting it into 96‐well plates, three cells per extraction at 300 μL. The software application SkanIt was used to analyze the extracted materials at 417 nm, which is the wavelength of the color. This allowed for the measurement of the total amount of alkaloids in the sample, or the number of yellow pigments.

#### 2.5.2 Determination of Oxalate Content

The modified titration method outlined by Jimoh, Afolayan, and Lewu (2020) was used to determine the oxalate content. About 1 g of each plant sample was diluted into 75 mL of 3 M H_2_SO_4_. The mixture was then stirred for an hour using a magnetic stirrer. After the mixture was filtered, 25 mL of the filtrate was heated to roughly 90°C. The hot aliquot was continuously titrated against 0.05 M KMnO4 until a 15‐s period of bright pink color shift was noticed, which marked the endpoint of titration. Each plant sample extract's titre value was multiplied by 2.2 mg of oxalate, which was calculated to be the equivalent of 0.05 M of KMnO4 utilized in the titration.

#### 2.5.3 Determination of Saponin Concentration

A 50 mL of 20% aqueous ethanol was mixed with five grams of powdered plant samples each to assess the percentage saponin content. Following a 30‐min orbital shaker, the mixture was heated for 4 h at 55°C in a water bath. Following filtering, the mixture was again extracted with residue using 200 mL of ethanol. In a water bath set at 90°C, the filtrates from both extractions were collected in a calibrated beaker and concentrated to roughly 40 mL. After shaking and decanting the concentrated filtrate into a separating funnel, 20 mL of diethyl ether was added and vigorously shaken. In the separating funnel, the mixture was let to settle until the ether and aqueous layers separated. The aqueous (bottom) layer was retained in the collecting beaker while the ether (upper) layer was disposed of. After reintroducing the set‐aside layer into the separating funnel, 60 mL of n‐butanol was added. After giving the mixture a good shake, it was allowed to settle until two different layers were visible. The top layer, which included butanol extract was collected and evaporated to a constant dry weight in an oven preheated to 40°C, while the lower layer was disposed away. The following equation was used to calculate the percentage saponin content of the sample.

$$\%\text{Saponin content}=\frac{\text{Weight of concentrated residue}}{\text{original weight of the sample}}\times 100$$

#### 2.5.4 Statistical Analysis

Upon the conclusion of the experiments, the data acquired from the treatments was expressed as means ± standard errors (SE) of three replicates. One‐way ANOVA and Fisher's least significant difference (LSD) were employed to compare the significant differences between treatment means at *p* < 0.05 using the MINITAB 17 statistical software.

## 3 Results

### 3.1 Effects of Salinity of the Mineral Composition

#### 3.1.1 Macronutrients

The results obtained from this study revealed that salinity concentrations significantly (*p* ≤ 0.05) influenced the macronutrient composition of flower buds, leaves, and roots of *Trachyandra ciliata*. From the results displayed in Table 1, the highest nitrogen yield (3890 mg/100 g) was recorded in the leaves under the control treatment, while the lowest mean values were recorded in the roots under high salinity treatments (150 and 200 mM). The highest Phosphorus content (450 mg/100 g) was recorded in both flower buds and the roots under control and 50 mM treatments, respectively, while the lowest Phosphorus yield was obtained in the samples collected from the leaves under high salinity treatment. Potassium content decreased indirectly proportional to increasing salinity among all plant parts. However, leaves followed by flower buds recorded considerably higher values compared to the roots. Interestingly, Calcium and magnesium recorded the highest mean values under 50 mM salinity in the flower buds, while low values were recorded under high salinity concentrations among all plant parts. Unsurprisingly, the Sodium content was recorded under high NaCl treatments, while control recorded the lowest Sodium content in all plant parts.

**TABLE 1:** The influence of varying salinity levels on the macronutrient composition of flower buds, leaves, and roots of *T. ciliata* .

Plant parts	NaCl conc.	N (mg/100g)	P (mg/100g)	K (mg/100 g)	Ca (mg/100g)	Mg (mg/100g)	Na (mg/100g)
Flower buds	0 mM	2700 ± 0.11d	450 ± 0.05a	3150 ± 0.05c	2620 ± 0.01cd	350 ± 0.01bc	220 ± 0.01gh
	50 mM	3320 ± 0.14b	390 ± 0.02b	2640 ± 0.14d	3680 ± 0.3a	550 ± 0.05a	2300 ± 0.2ef
	100 mM	2440 ± 0.06de	360 ± 0.01b	2150 ± 0.05ef	2410 ± 0.1de	270 ± 0.01def	2800 ± 0.3de
	150 mM	—	—	—	—	—	—
	200 mM	—	—	—	—	—	—
Leaves	0 mM	3890 ± 0.16a	380 ± 0.03b	6770 ± 0.27a	2530 ± 0.46d	320 ± 0.02bcde	720 ± 0.17g
	50 mM	3010 ± 0.07c	360 ± 0.02b	4840 ± 0.08b	3210 ± 0.11ab	370 ± 0.02b	3730 ± 0.1c
	100 mM	3000 ± 0.23c	250 ± 0.01c	3420 ± 0.19c	1790 ± 0.13fg	310 ± 0.01cde	4830 ± 0.71b
	150 mM	2170 ± 0.07efg	180 ± 0.01d	2470 ± 0.13de	1600 ± 0.01fg	270 ± 0.02efg	7910 ± 0.17a
	200 mM	2050 ± 0.05gh	120 ± 0.01e	1810 ± 0.12fg	1310 ± 0.02g	210 ± 0.01h	8530 ± 0.22a
Roots	0 mM	2070 ± 0.06fgh	380 ± 0.02b	1700 ± 0.18g	3150 ± 0.33abc	330 ± 0.03bcd	450 ± 0.05 gh
	50 mM	2340 ± 0.04ef	450 ± 0.02a	1590 ± 0.03g	3210 ± 0.05ab	270 ± 0.03efg	1760 ± 0.23f
	100 mM	2040 ± 0.02gh	340 ± 0.01b	930 ± 0.02hi	2790 ± 0.08bcd	220 ± 0.01fgh	2690 ± 0.03e
	150 mM	1830 ± 0.03hi	280 ± 0.01c	620 ± 0.03i	1920 ± 0.03ef	210 ± 0.02gh	3010 ± 0.1de
	200 mM	1610 ± 0i	230 ± 0.02cd	1030 ± 0.01h	1890 ± 0.2ef	120 ± 0.01i	3490 ± 0.18cd

*Note:* Values (mean ± SE) in each column followed by the different letters are significantly different at *p* ≤ 0.05 (*). Dash (—) = No flower buds produced.

Abbreviation: ns, not significantly different.

#### 3.1.2 Micronutrients

Table 2 shows that varying NaCl concentrations significantly affected the micronutrient contents of flower buds, leaves, and roots at *p* ≤ 0.05. Surprisingly, the flower buds recorded the lowest Manganese (Mn) content when compared to other parts of the plant. The highest composition was obtained in the leaves under 100 mM treatment, while roots maintained an average Mn content. On the contrary, the highest Iron (Fe) mean values were recorded in the roots, with control treatment recording the highest Fe content (46.5 mg/100 g), while the lowest values were recorded in the flower buds. The accumulation of other heavy metals (Copper and Zinc) was directly proportional to increasing salinity in all plant parts. The highest Cu mean values (7.9 and 3.15 mg/100 g) were recorded in the roots under 200 and 100 mM treatments, respectively, while the lowest value was recorded in the flower buds under control treatment. Similarly, the highest Zn content (70.05 and 51.9 mg/100 g) was recorded in the roots under 200 mM and 100 mM salinity concentrations, respectively, while control treatment in the flower buds recorded the lowest Zn content (2.85 mg/100 g).

**TABLE 2:** The influence of varying salinity levels on the micronutrient composition of flower buds, leaves, and roots of *T. ciliata* .

Plant parts	NaCl conc.	Mn (mg/100 g)	Fe (mg/100 g)	Cu (mg/100 g)	Zn (mg/100 g)
Flower buds	0 mM	1.95 ± 0.05 g	10.8 ± 0.6efg	0.25 ± 0.05 hij	2.3 ± 0.1gh
	50 mM	2.2 ± 0.1 g	9.4 ± 0.5 fg	0.35 ± 0.05ghi	2.55 ± 0.05gh
	100 mM	2.35 ± 0.05 g	6.25 ± 0.35 gh	0.55 ± 0.05 fg	2.85 ± 0.05g
	150 mM	—	—	—	—
	200 mM	—	—	—	—
Leaves	0 mM	8.8 ± 0.1b	10.5 ± 1.7efg	0.1 ± 0.02ij	2.7 ± 0.3gh
	50 mM	9.2 ± 0.3b	12.6 ± 2.3ef	0.15 ± 0.03hij	3.25 ± 0.35g
	100 mM	11.7 ± 0.2a	30.4 ± 1.8c	0.25 ± 0.05 hij	4.05 ± 0.15g
	150 mM	8.65 ± 0.15b	22.7 ± 0.6d	0.4 ± 0.06fgh	7.45 ± 1.25f
	200 mM	5.85 ± 0.25d	16.7 ± 0.3de	0.65 ± 0.05f	23.9 ± 0.3e
Roots	0 mM	4.55 ± 0.75e	46.5 ± 7a	1.65 ± 0.25e	27.6 ± 0.15d
	50 mM	4.75 ± 0.55e	30.15 ± 1.05c	2 ± 0.05d	37.8 ± 2.8c
	100 mM	7.1 ± 0.2c	37.4 ± 1.4b	3.15 ± 0.05b	51.9 ± 1.1b
	150 mM	5.2 ± 0.1de	32.65 ± 0.65bc	2.75 ± 0.05c	37.45 ± 0.45c
	200 mM	3.7 ± 0.1f	38.9 ± 1b	7.9 ± 0.1a	70.05 ± 1.05a

*Note:* Values (mean ± SE) in each column followed by the different letters are significantly different at *p* ≤ 0.05 (*). Dash (—) = No flower buds produced.

Abbreviation: ns, not significantly different.

### 3.2 Influence of Salinity on the Proximate Content

This study discovered that varying salinity led to significant variations on the nutritional contents of flower buds, leaves, and roots of *Trachyandra ciliata* with regards to NDF, moisture, fat, ash, and protein (Table 3). From the results gathered in this study, moisture content decreased with increasing salinity in all plant parts. The highest moisture content was recorded on the samples collected from the leaves under control treatment, 50 mM and 100 mM, respectively, followed by the roots under control and low salinity, while the lowest moisture content was recorded in the flower buds under high salinity treatment. On the other hand, the ash content increased with increasing salinity with the highest comparable values (36.31% and 33.67%) obtained in the leaves under 200 mM and 150 mM treatments, respectively. Similarly, flower buds recorded the lowest ash content compared to other plant parts, with the lowest mean value (9.4) recorded under control treatment. The highest protein content (24.34%) was recorded in the leaves under control treatment followed by 20.73% recorded in the flower buds under 50 mM salinity. The lowest protein content (10.08%) was obtained from the samples collected from the roots under 200 mM, although value was comparable to 11.46% recorded in the roots under 150 mM salinity treatment. Interestingly, the highest NDF content (39.45%) was recorded on the samples collected from the flower buds under low salinity treatment, although this was comparable to the mean value obtained the flower buds under control treatment. The lowest NDF content was recorded in the roots under 200 mM salinity treatment, although it was comparable to the values obtained from 100 and 150 mM in the roots, 100 mM in the flower buds, and all values obtained from the leaves.

**TABLE 3:** The influence of varying salinity levels on the proximate content of flower buds, leaves, and roots of *T. ciliata* .

Plant parts	NaCl conc.	Moisture %	Ash %	Fat %	Protein %	NDF %
Flower buds	0 mM	5.24 ± 0.09cde	9.4 ± 0.7 g	2.75 ± 0.05b	16.87 ± 0.67d	35.72 ± 0.02ab
	50 mM	4.67 ± 0.06ef	11.37 ± 0.47fg	3.1 ± 0.11a	20.73 ± 0.88b	39.45 ± 0.95a
	100 mM	3.3 ± 0.1 g	12.83 ± 0.28f	2.05 ± 0.15de	15.25 ± 0.35de	29.66 ± 1.32de
	150 mM	—	—	—	—	—
	200 mM	—	—	—	—	—
Leaves	0 mM	7.15 ± 0.25a	28.06 ± 0.1bc	2.46 ± 0.04c	24.34 ± 0.97a	30.39 ± 2.26de
	50 mM	6.16 ± 0.26b	30.02 ± 0.82b	2.77 ± 0.03b	18.84 ± 0.46c	28.51 ± 0.34de
	100 mM	6.12 ± 0.25b	29.96 ± 1.24b	2.34 ± 0.03c	18.77 ± 1.46c	29.39 ± 0.19de
	150 mM	5.14 ± 0.24cde	33.67 ± 0.43a	2.34 ± 0.14c	13.55 ± 0.45efg	29.33 ± 0.43de
	200 mM	4.35 ± 0.59f	36.34 ± 0.96a	2.26 ± 0.11cd	12.83 ± 0.31gh	27.62 ± 0.32de
Roots	0 mM	5.75 ± 0.18bc	24.06 ± 3.5d	1.72 ± 0.08f	12.94 ± 0.35fgh	35.22 ± 2.07abc
	50 mM	5.4 ± 0.17cd	18.77 ± 1.55e	1.8 ± 0.2ef	14.65 ± 0.27ef	31.57 ± 0.83bcd
	100 mM	4.82 ± 0.05def	24.76 ± 0.06cd	1.62 ± 0.07f	12.77 ± 0.14gh	28.87 ± 4.67de
	150 mM	4.75 ± 0.05def	25.96 ± 0.66cd	1.6 ± 0.08f	11.46 ± 0.16hi	30.66 ± 1.36cde
	200 mM	4.3 ± 0.38f	30.06 ± 0.15b	1.29 ± 0.08 g	10.08 ± 0.01i	26.55 ± 0.35e

*Note:* Values (mean ± SE) in each column followed by the different letters are significantly different at *p* ≤ 0.05 (*). Dash (—) = No flower buds produced.

Abbreviation: ns, Not significantly different.

### 3.3 Effects of Salinity on the Antinutrient Content

The antinutrient composition of flower buds, leaves, and roots of *T. ciliata* cultivated under varying salinity concentrations is presented in Table 4 as a percentage of oxalate, saponin, and alkaloid in the crude extract. The study discovered that salinity has a significant influence on the antinutrient composition of the flower buds, leaves, and roots of wild cabbage. All antinutrient parameters increased with increasing salinity across all plant parts. The roots under 200 mM salinity treatment recorded the highest oxalate content (4.02%) followed by 3.59% which was also obtained in the roots under 150 mM salinity treatment, although this value was statistically comparable to 3.55% that was obtained in the flower buds under 100 mM salinity treatment. The lowest oxalate content (1.83%) was obtained in the leaves under 50 mM salinity level. On the other hand, the highest saponin content (5.7%) was recorded in the samples obtained from the leaves under 200 mM salinity followed by 5.07% which was also recorded in the leaves under 150 mM, although this value was statistically comparable to 4.77% that was obtained in the flower buds under 100 mM salinity treatment. The lowest saponin content (0.91%) was recorded in the samples collected from the leaves under control treatment. Alkaloid composition of wild cabbage was relatively lower compared to other parameters, with the highest value (0.41) obtained in the roots under 200 mM, while the lowest value (0.02%) was recorded in the flower buds under control treatment.

**TABLE 4:** The influence of varying salinity levels on the antinutrient composition of flower buds, leaves, and roots of *T. ciliata* .

Plant parts	NaCl conc.	Oxalate %	Saponin %	Alkaloids %
Flower buds	0 mM	2.12 ± 0.06fgh	2.28 ± 0.08g	0.02 ± 0.005ef
	50 mM	2.67 ± 0.25d	3.29 ± 0.67f	0.03 ± 0.005ef
	100 mM	3.55 ± 0.11b	4.77 ± 0.1bc	0.08 ± 0.01de
	150 mM	—	—	—
	200 mM	—	—	—
Leaves	0 mM	1.86 ± 0.05h	3.89 ± 0.01e	0.04 ± 0.02def
	50 mM	1.83 ± 0.12h	4.16 ± 0.03de	0.03 ± 0.001ef
	100 mM	2.1 ± 0.11gh	4.39 ± 0.02cde	0.07 ± 0.01de
	150 mM	2.49 ± 0.02de	5.07 ± 0.13b	0.1 ± 0.01d
	200 mM	2.43 ± 0.21def	5.7 ± 0.1a	0.18 ± 0.03c
Roots	0 mM	2.19 ± 0.05efg	0.91 ± 0.61h	0.05 ± 0.01def
	50 mM	3.07 ± 0.05c	2.07 ± 0.04g	0.05 ± 0.005def
	100 mM	3.37 ± 0.08bc	3.13 ± 0.02f	0.11 ± 0.03d
	150 mM	3.59 ± 0.08b	3.2 ± 0.01f	0.28 ± 0.07b
	200 mM	4.02 ± 0.06a	4.51 ± 0.16cd	0.41 ± 0.02a

*Note:* Values (mean ± SE) in each column followed by the different letters are significantly different at *p* ≤ 0.05 (*). Dash (—) = No flower buds produced.

Abbreviation: ns, Not significantly different.

### 3.4 Effects of Salinity on the Phytochemical and Antioxidant Composition

The phytochemical and antioxidant capacity of flower buds, leaves, and roots of wild cabbage grown under different salinity levels is displayed in Table 5 in the form of polyphenols, flavonols, Ferric Reducing Ability of Plasma (FRAP), 2,2′‐Azinobis (3‐ethylbenzothiazoline‐6‐Sulfonic Acid (ABTS), and 2,2‐Diphenyl‐1‐Picrylhydrazyl (DPPH). The results obtained from this study reveal that different salinity concentrations had a significant influence on the phytochemical and antioxidant capacity of flower buds, leaves, roots of wild cabbage at *p* ≤ 0.05. Polyphenolic content increased in direct proportion to increasing salinity across all plant parts. The highest polyphenol (15.37 mg QE/L) was recorded in the roots under 150 mM salinity level followed by 13.32 mg QE/L recorded in the roots under 200 mM salinity, although this amount was not significantly different from 12.93 mg QE/L that was recorded in the flower buds under 100 mM salinity treatment. The lowest polyphenol content was recorded in the samples cultivated under control and low salinity in the leaves. On the other hand, the highest flavonol composition (3.78 and 3.69 mg QE/L) was recorded in the leaves under 200 mM and 150 mM, respectively, while the lowest value (1.56 mg mg QE/L) was recorded in the samples collected from the roots under control treatment. The highest FRAP activity was observed in the roots followed by flower buds, while leaves recorded the lowest FRAP activity. The highest FRAP activity (105.41 μmol AAE/g) was recorded on the samples cultivated under 150 mM in the roots, while the lowest activity (40.65 μmol AAE/g) was observed under control treatment in the leaves. Interestingly, the highest ABTS and DPPH scavenging activities were both recorded in the samples cultivated under 200 mM salinity in the roots, followed by 150 mM salinity in the roots, while they both had the lowest antioxidant activities under samples collected from the leaves under control treatment.

**TABLE 5:** The influence of varying salinity levels on the phytochemical and antioxidant capacity of flower buds, leaves, and roots of *T. ciliata* .

Plant parts	NaCl conc.	Polyphenols (mg GAE/g)	Flavonols (mg QE/L)	FRAP (μmol AAE/g)	ABTS (μmoL TE/g)	DPPH (μmoL TE/g)
Flower buds	0 mM	9.57 ± 0.59f	2.3 ± 0.06d	61.78 ± 1.25e	38.54 ± 1.32e	25.05 ± 0.67e
	50 mM	12.07 ± 0.27d	2.77 ± 0.04c	65.49 ± 2.25e	39.43 ± 1.04de	29.95 ± 0.99d
	100 mM	12.93 ± 0.15bc	3.3 ± 0.14b	73.91 ± 1.41d	47.96 ± 0.24b	30.18 ± 1.37d
	150 mM	—	—	—	—	—
	200 mM	—	—	—	—	—
Leaves	0 mM	8.18 ± 0.18g	2.17 ± 0.13de	40.66 ± 0.34h	31.56 ± 0.32h	12.86 ± 0.9h
	50 mM	8.67 ± 0.02g	2.92 ± 0.04c	43.52 ± 0.29gh	32.17 ± 0.3h	15.86 ± 0.71gh
	100 mM	9.79 ± 0.08f	3.32 ± 0.07b	45.52 ± 0.31g	32.93 ± 0.12gh	17.09 ± 0.86g
	150 mM	10.18 ± 0.18f	3.69 ± 0.06a	47.1 ± 0.37g	33.96 ± 0.09 g	19.05 ± 0.33fg
	200 mM	11.43 ± 0.29e	3.78 ± 0.15a	54.4 ± 1.07f	36.38 ± 0.67f	21.53 ± 0.27f
Roots	0 mM	8.38 ± 0.16g	1.56 ± 0.09 g	66.11 ± 2.05e	36.48 ± 0.71f	32.78 ± 0.36cd
	50 mM	12.39 ± 0.13cd	1.96 ± 0.03ef	79.8 ± 2.99c	40.59 ± 0.7d	34.88 ± 1.34c
	100 mM	13 ± 0.1bc	2.37 ± 0.09d	86.8 ± 0.9b	42.8 ± 0.54c	35.74 ± 0.62c
	150 mM	15.37 ± 0.08a	2.75 ± 0.07c	105.41 ± 0.69a	47.03 ± 0.59b	41.52 ± 2.86b
	200 mM	13.32 ± 0.24b	1.82 ± 0.08f	83.64 ± 3.22bc	61.93 ± 0.35a	56.48 ± 1.78a

*Note:* Values (mean ± SE) in each column followed by the different letters are significantly different at *p* ≤ 0.05 (*). Dash (—) = No flower buds produced.

Abbreviation: ns, Not significantly different.

## 4 Discussion

Several studies have been conducted on the effects of salinity on halophytes, with results suggesting that salinity modulates growth, phytochemical, proximate, and mineral composition of different halophyte species (Bulawa et al. 2022; Mndi et al. 2023; Sogoni et al. 2021; Tshayingwe et al. 2023). However, developments in the food science sector and consumer preferences for varied diets also corroborate the consumption of wild edible halophytic plants as diet complements as well as nutritious and useful foods for specific conditions (Petropoulos et al. 2018). *Trachyandra ciliata* is one of the underutilized halophytes from South Africa. There is paucity of information on its nutrition and edibility even though its flower buds were reportedly consumed by the Khoisan people that lived in the western Cape coast of South Africa (Smith and Van Wyk 1998). Investigating the proximate, antinutrient, mineral, and phytochemical composition of *T. ciliata* cultivated under different levels of salinity became crucial to validate its edibility and promote its consumption and commercialization.

In this study, varying salinity concentrations caused significant variations in the mineral, proximate, antinutrient, and phytochemical composition of *T. ciliata* . A significantly higher macro and micronutrient content was recorded in the flower buds and leaves of the untreated (control) plants and those cultivated with 50 mM treatments although heavy metals such as Zn and Cu increased with increasing salinity and significantly higher in the roots. The decrease in macro and micronutrient content with increasing salinity can be attributed to osmotic stress, decreased water availability, changes in the cellular ionic balance, and excessive presence of Na^+^ and Cl^−^ that are caused by salinity, which may lead to deficiency and or toxicity of some nutrients (Ehtaiwesh 2022). The stability of nutrients under low salinity treatments is caused by the ability of halophytes to maintain osmotic balance, cell homeostasis, and nutrient balance under salinity stress (Corwin 2021; Shabala and Pottosin 2014). The increase and stability in some micronutrients may be caused by the ability of halophytic plants to employ morphophysiological strategies such as salt exclusion, salt excretion via specialized organs like salt glands or salt bladders, and by diluting salt ions through succulence (Rasouli et al. 2021; Shabala 2013). These strategies contribute to the ability of halophytes to maintain cell balance under salinity stress.

Minerals are essential parts of the human diet. They sustain life by supplying the essential nutrients required for the body's psychophysical well‐being (Jimoh, Afolayan, and Lewu 2020). Most of the mineral composition of the investigated parts of *T. ciliata* are similar to that of the flower buds of a closely related species *T. divaricata* , (Bulawa et al. 2022; Tshayingwe et al. 2023) although they exceeded the Recommended Daily Allowance (RDA). For instance, the calcium content of *T. ciliata* is well above the amounts recorded previously on the flower buds of a closely related *T. divaricata* that had the highest value of 1101 mg/100 g and 700 mg/100 g (Bulawa et al. 2022; Tshayingwe et al. 2023). Calcium has an RDA of 1000 mg for an adult (Vannucci et al. 2017), which was exceeded in all examined samples regardless of plant part or salinity treatment. This suggests that frequent consumption of wild cabbage will promote healthy bones, muscles, and may limit the chance of osteopenia and osteoporosis in cancer patients as calcium is essential for the development and maintenance of bone and muscle (Tu et al. 2016).

Potassium (K) is a necessary ingredient for physiological function because extracellular and intracellular cations are needed to sustain blood pressure, nerve impulse conduction, and muscle contraction (Udensi and Tchounwou 2017). The recommended consumption of K is approximately 2000 mg for an adult, which is widely present in wild cabbage. In the current study, all the samples collected from flower buds and leaves exceeded the minimum RDA of K except the roots samples which did not meet the RDA. These results corroborate earlier findings on other halophytes such as *T. divaricata* , *Tetragonia decumbens*, and *Mesembryanthemum crystallinum* where the potassium content exceeded the RDA values (Bulawa et al. 2022; Mndi et al. 2023; Sogoni et al. 2021). In addition, most of the samples examined in this study have higher K content than the edible part of *Asparagus officinalis* (2760 mg/100 g) which resembles the flower bud of *T. ciliata* (Redondo‐Cuenca et al. 2023). The reduction in K content of the root samples may be related to high ionic imbalances caused by an excess of Na^+^ and CI^−^ that may reduce the selectivity of root membranes (Avarseji et al. 2013; Botella et al. 1997; Nieves‐Cordones, Al Shiblawi, and Sentenac 2016).

Magnesium (Mg) performs several essential functions, such as stabilizing Ca/K homeostasis, releasing neurotransmitters, playing structural roles in proteins and polyribosomes, cell adhesion, nucleic acids, and enzyme cofactors (Cebani et al. 2024; Ksouri et al. 2011). In the present study, Mg content was found to be abundant in wild cabbage and well above the RDA. For an example, the USDA recommended 12 mg for cooked broccoli and 55 mg for amaranth food as standard magnesium content for 100 mg, which is far below the findings of this study (Drewnowski et al. 2022). The Mg content in tested plant samples ranged from 120 to 550 mg/100 g, suggesting that wild cabbage is very rich in Mg when subjected to salinity. These findings concur with the results of Tshayingwe et al. (2023) on *T. divaricata* where Mg content ranged from 249.00 to 478.50 mg/100 g. Similarly, findings from this study are also comparable to the data provided by Redondo‐Cuenca et al. (2023) on the edible portion of *Asparagus* in which a magnesium level of 210 mg/100 g was reported. In contrast to the Mg content, the phosphorus content of all the samples was below the RDA of 700 mg. The highest phosphorus content (450 mg/100 g) was recorded in the flower buds under the control treatment. Phosphorus is necessary for human nutrition since it serves as a physiological buffer, a substrate for vital cellular processes, and a part of bone mineral in the skeleton (Chang and Anderson 2024). These results are in contradiction with the reports on *T. divaricata* , a closely related species of *T. ciliata* , in which flower buds met the RDA (Bulawa et al. 2022; Tshayingwe et al. 2023). The reduction in phosphorus may be related to findings from studies of Benito et al. (2014) and Serna and Bergwitz (2020) which suggest that excessive presence of Na^+^ in the plant cells negatively affects plant absorption, mobility, and bioavailability of phosphorus. Furthermore, this reduction in phosphorus level corresponds to the findings on a South African halophyte *T. decumbens* , in which increasing NaCl levels reduced the phosphorus and other macronutrient composition in a stable manner. Notably, Na^+^ increased with increasing salinity due to NaCl application, although it was notably higher in the leaves than other plant parts tested. The high Na content in the leaves is consistent with recent findings that *T. ciliata* employs different physiological mechanisms in the leaves such as excretion in the form of salt crystals and the formation of salt‐secreting glands that aid bioaccumulation and storage of excessive salts (Ngxabi et al. 2024). These results on the macronutrient composition suggest that wild cabbage can maintain cell homeostasis and optimum biochemical processes under low salinity compared to higher salinity levels (Shabala 2013).

The critical micronutrients such as Iron (Fe), Manganese (Mn), Copper (Cu), and Zinc (Zn) are all required by the body in smaller quantities (less than 20 mg per day) and constitute less than 0.01% of the total body weight (National Research Council 1989; Ogundola, Bvenura, and Afolayan 2018). Most of the samples examined in this study recorded micronutrient contents far less than the RDA, except for zinc in the roots where all treatments recorded higher values than the RDA. These results agree with the findings of Bulawa et al. (2022) and Sogoni et al. (2021) who, respectively, reported micronutrients far below the RDA in *T. divaricata* and *Tetragonia decumbens* both of which are related edible halophytes from the same region. Nevertheless, these findings support the reports on edible *Asparagus officinalis* shoots which resembles the edible flower bud of *T. ciliata* (Redondo‐Cuenca et al. 2023). Moreover, the low accumulation of heavy metal by wild cabbage is in line with earlier reports which suggested that sufficient uptake and transportation of K^+^ under saline conditions contribute to mitigating ion toxicity in plant cells (Benito et al. 2014; Sogoni et al. 2021; Wang et al. 2023). It could be inferred from this study that *T. ciliata* can maintain cell balance and optimum micronutrient ratios under high salinity conditions. Hence, it can be assumed that wild cabbage is safe for consumption and may be used as an alternative to the well‐known *Asparagus*.

Increasing salinity caused variations in the proximate content (Moisture, ash, protein, NDF, and fat) of wild cabbage. The moisture content was significantly higher in all plant parts cultivated under low salinity treatments while higher concentrations recorded lower moisture contents. However, the moisture content of the tested samples ranged from 3.3% to 7.15%, implying that this species may have lower microbial contamination and chemical degradation, which is usually related to high moisture content (Mndi et al. 2023; Ooi, Iqbal, and Ismail 2012). On the contrary, higher salinity concentrations increased ash content in all plant parts. One of the main characteristics of halophytes is their high ash concentration. Therefore, the primary factor that limits the use of halophytes in uncooked dishes may be their high ash concentration (Wang et al. 2023). Ash content in the present study ranged from 9.4% to 36.34%, which is relatively comparable to the composition of most processed foods (Redondo‐Cuenca et al. 2023). However, the ash content of the edible flower bud of *T. ciliata* ranged from 9.4% to 12.83%, which is significantly lower than processed foods. Tshayingwe et al. (2023) reported similar data on the inflorescence of a closely related species ( *T. divaricata* ). A similar trend was reported by Hessini et al. (2020) on Chenopodiaceae species where increasing salinity led to increased ash content and further suggested that high ash content in halophytes is a major limiting factor on nutrition and use. However, a high ash value suggests that the plant contains significant quantities of dietary fiber which harbors and protects digestive organisms in the gastrointestinal tract (Hessini et al. 2020; Redondo‐Cuenca et al. 2023).

Along with other nutrients, the protein content plays a significant role in a variety of processes, including signal transduction, osmotic and ionic homeostasis, photosynthesis, and reactive oxygen species‐scavenging systems (Cebani et al. 2024; Mndi et al. 2023; Wang et al. 2023). In the present study, crude protein was significantly higher under low salinity treatments and decreased with increasing salinity. These results are in correspondence with earlier studies on other halophytes, where protein content increased under low salinity and decreased with increasing salinity in the irrigation water (Sogoni et al. 2021; Wang et al. 2023). The initial crude protein increase is an important component of salt tolerance. As such, there is a general trend for transporter protein activity and abundance to increase under hyperosmotic salinity at first and then tend to decrease as the concentration of salinity in the soil and irrigation water increases (Koyro et al. 2013). According to Koyro et al. (2013), a decrease in crude protein content is a sign of extreme stress and eventually lead to plant death, which is in line with the current findings. Low availability of amino acids, denaturation of enzymes involved in protein synthesis, and decreased nutrient uptake from saline soils are indicators and potential causes of decreased crude protein concentration in saline environments (Hedayati‐Firoozabadi et al. 2020; Wang et al. 2023).

In most instances, wild vegetables including edible halophytes have previously been reported to contain relatively low unsaturated fat content, ranging from 2% to 4% (Mndi et al. 2023). The crude fat content of the samples in the present study concurs with previous studies on South African edible halophyte species, *Mesembryanthemum crystallinum* and *Trachyandra divaricata*, where crude fat did not exceed 4% (Bulawa et al. 2022; Mndi et al. 2023). Foods high in fat can raise cholesterol, which is a major contributor to cardiovascular diseases (Wang et al. 2023). As a result, eating this green vegetable will help manage weight loss and ailments brought on by an excess of fat. Dietary fiber is necessary to reduce the absorption of cholesterol, prevent cardiovascular disease, and regulate bowel motions (Carlsen and Pajari 2023; Nie and Luo 2021). In the present study, the NDF ranged from 26.55% to 39.45%. This was higher than NDF values that have been reported previously in other underutilized vegetables such as *M. crystallinum* (29%), *Solanum nigrum* (9.56%), *Amaranthus cruentus* (8.45%), and *Celosia argentea* (23%) (Adegbaju, Otunola, and Afolayan 2019; Ajayi et al. 2018; Mndi et al. 2023). The high NDF concentration in *T. ciliata* suggests that the species may help control intestinal transit, boost dietary bulk, and reduce the risk of numerous metabolic diseases orchestrated by inadequate consumption of crude fiber, including colon cancer, obesity, and diabetes (Adegbaju, Otunola, and Afolayan 2019).

Contrary to nutritious elements, antinutrients temper with the absorption of some essential minerals and micronutrients in the digestive system which might have a harmful impact on the functioning of certain organs (Adegbaju, Otunola, and Afolayan 2019; Jimoh, Afolayan, and Lewu 2020). In this study, increasing salinity caused significant variations on the accumulation of three antinutrients, namely, alkaloids, oxalic acids, and saponins in all plant parts. Alkaloids are the active substances in many medicinal plants, which are naturally occurring chemical compounds containing basic nitrogen atoms (Gemede and Ratta 2014). However, a high alkaloid accumulation is poisonous to both people and animals (Matsuura and Fett‐Neto 2017). The alkaloid concentration recorded in the current study is far below the concentration that is perceived to be toxic, making the alkaloid content of the plant more of pharmacological importance than toxicity (Ajayi et al. 2018). Reports from previous studies suggest that excessive levels of saponins can inhibit digestive and metabolic enzymes and bind with minerals like zinc, which can impact nutritional absorption (Ajayi et al. 2018; Wang et al. 2023). The saponin concentrations recorded in the current study ranged from 2.28% to 5.7%, which is comparable to the results reported on *Celosia argentea* with saponin content of 5.3% (Adegbaju, Otunola, and Afolayan 2019). Notwithstanding, it is believed that saponin content lower than 10% in a diet is harmless to the human body (Adegbaju, Otunola, and Afolayan 2019; Gemede and Ratta 2014). This suggests that saponin level of *T. ciliata* is within the harmless concentration and may be considered safe for consumption. Oxalate is an antinutrient that is contained in the sap of many green leafy vegetable crops. It works by creating a powerful chelate with calcium and other minerals found in food, which prevents the body from absorbing and assimilating those nutrients (Gemede and Ratta 2014; Natesh, Abbey, and Asiedu 2017). Kidney stones are a major health issue caused by the accumulation of this insoluble calcium oxalate in crystal form within the kidney (Natesh, Abbey, and Asiedu 2017). In the present study, the highest oxalate concentration was recorded under high salinity treatment in all plant parts, with the highest value (4.02%) recorded in the root samples. However, the highest values for oxalate composition of *T. ciliata* in all plant parts are lower than what was recorded in the previous studies for *Celosia argentea* (4.85%), *Amaranthus cruentus* (5.08%), and *Solanum nigrum* (5.88%) (Adegbaju, Otunola, and Afolayan 2019; Ajayi et al. 2018). The low antinutrient composition of wild cabbage further confirms its edibility and safety for human consumption.

To mitigate the consequences of oxidative stress, plants accumulate secondary metabolites, including phenolic compounds that function as hydrogen donors, reducing agents and singlet oxygen quenchers (Ogbe, Finnie, and Van Staden 2020). A plant‐based diet rich in phenolic content can prevent oxidative damage to human tissue by scavenging free radicals and preventing lipid peroxidation. This improves nutritional quality and helps prevent issues that may arise from consuming too many synthetic additives (Tshayingwe et al. 2023). Therefore, it is important to apply techniques that improve phytochemical properties of vegetables during cultivation. Furthermore, phenolic compounds are of great importance due to their significant impact on the flavor and taste of food products, which has given high interest to the screening of plants for these compounds (Cebani et al. 2024; Mndi et al. 2023). In the current study, phenolic compounds significantly increased in direct proportion with increasing salinity concentration. The flower buds accumulated the highest phenolic compounds under moderate salinity (100 mM). On the other hand, leaves and roots accumulated highest phenolic content under high salinity treatments (150 mM and 200 mM). These findings correspond to recent studies on other halophytes (*Schizonepeta tenuifolia* and *Tetragonia decumbens*), which reported that increasing salinity enhanced the accumulation of phenolic compounds (Sogoni et al. 2021; Zhou et al. 2018). A similar trend was reported for other species cultivated under varying salinity, where increasing salinity significantly increased the accumulation of phenolic compounds and antioxidant capacities of *Cakile maritima* , *Cynara scolymus* and *Matthiola incana* (Jafari and Hashemi Garmdareh 2019; Ksouri et al. 2007; Rezazadeh et al. 2012).

## 5 Conclusions

For the first time, this study validates the edibility of *T. ciliata* based on the high accumulation of proximate and mineral contents of the plant as stated in earlier reports that lacked scientific justification. Its high fiber, protein and energy content, particularly in the flower buds, confirms that it is effective for human digestion, and could serve as an immune system booster, a significant source of nutrition, and as a functional food. From the results obtained in this study, low salinity has shown to be more effective for maximum production of nutritional content. This opens the potential of wild cabbage to be cultivated in abandoned saline soils or diluted seawater, which is a water‐saving approach. The low antinutrient composition and high phenolic content of the plant reflect its nutraceutical potential and safety to be consumed by humans. Therefore, *T. ciliata* is recommended for domestication and commercial cultivation. Similar studies are recommended on other wild edible plants to mitigate the looming food shortage and hidden hunger due to increasing population and water scarcity.

## Author Contributions

**Sihle Ngxabi:** conceptualization (equal), data curation (equal), formal analysis (equal), investigation (equal), methodology (equal), visualization (equal), writing – original draft (equal), writing – review and editing (equal). **Muhali Olaide Jimoh:** conceptualization (equal), formal analysis (equal), software (lead), supervision (equal), validation (equal), visualization (equal), writing – review and editing (equal). **Avela Sogoni:** investigation (supporting), methodology (supporting), software (equal), visualization (equal), writing – review and editing (equal). **Learnmore Kambizi:** conceptualization (equal), funding acquisition (lead), project administration (equal), resources (lead), supervision (equal), validation (equal), writing – review and editing (equal). **Charles Petrus Laubscher:** funding acquisition (supporting), project administration (equal), supervision (equal), validation (equal), writing – review and editing (equal). **Fanie Rautenbach:** data curation (equal), formal analysis (equal), methodology (equal), resources (equal), software (equal), writing – review and editing (equal).

## Ethics Statement

The authors have nothing to report.

## Conflicts of Interest

The authors declare no conflicts of interest.

## Data Availability

The data that support the findings of this study are available on request from the corresponding author.
